# Epigenetic regulation of HIV-1 transcription: insights into latency mechanisms and therapeutic strategies

**DOI:** 10.3389/fcimb.2026.1926141

**Published:** 2026-09-17

**Authors:** Shuhan Yang, Jinsong Yuan, Xiaodong Sun, Chen Huan

**Affiliations:** 1 Center of Infectious Diseases and Pathogen Biology, Institute of Virology and AIDS Research, Key Laboratory of Organ Regeneration and Transplantation of The Ministry of Education, The First Hospital of Jilin University, Changchun, Jilin, China; 2 Department of Hepatobiliary and Pancreatic Surgery, General Surgery Center, The First Hospital of Jilin University, Changchun, Jilin, China

**Keywords:** epigenetic regulation, histone modifications, HIV-1, HIV-1 latency, LTR, therapeutic strategies

## Abstract

HIV-1 is one of the most serious pathogens currently threatening human health worldwide. Although antiretroviral therapy (ART) effectively suppresses HIV-1 replication, it fails to eliminate the latent reservoir of transcriptionally silent proviruses that persist in long-lived cells, making HIV-1 latency the major obstacle to achieving a functional cure for AIDS. The establishment and reversal of HIV-1 latency are largely regulated by epigenetic regulatory mechanisms involving the viral long terminal repeat (LTR) promoter. Following proviral integration into the host genome, the viral DNA is rapidly assembled into chromatin, where nucleosome positioning and diverse epigenetic modifications regulate viral transcription activity. At the chromatin level, nucleosome positioning at the LTR contributes to constrained transcriptional accessibility. In addition, coordinated histone methylation and DNA methylation promote transcriptionally repressive states, whereas histone acetylation dynamics facilitate chromatin relaxation and transcriptional reactivation in response to cellular stimuli. Beyond these classical mechanisms, emerging studies highlight additional regulatory mechanisms, including histone crotonylation and non-coding RNA, which further regulate HIV-1 epigenetic control. These mechanisms constitute a multilayered regulatory network that enables both stability of latency and potential reactivation upon perturbation. These lines of evidence not only help elucidate the mechanisms underlying the establishment and maintenance of latency, but also support the development of effective strategies to target latent reservoirs. This review synthesizes current understanding of how chromatin organization and epigenetic regulation modulates the establishment, maintenance and reactivation of HIV-1 latency, and discusses prospective epigenetic-targeting strategies for latent reservoir elimination.

## 1 Introduction

Since the first cases of acquired immunodeficiency syndrome (AIDS) were reported in 1981, human immunodeficiency virus type 1 (HIV-1) infection has claimed over 40 million lives worldwide (World Health Organization, 2025). Despite remarkable advances in antiretroviral therapy (ART), which effectively suppress active viral replication, HIV-1 infection remains incurable due to the persistence of latent viral reservoirs. These reservoirs consist of long-lived cells containing transcriptionally silent but replication-competent proviruses, which can rapidly resume viral production after ART interruption and represent the primary obstacle to achieving an HIV cure (Finzi et al., 1997; Arts and Hazuda, 2012; Chinese guidelines for the diagnosis and treatment of human immunodeficiency virus infection/acquired immunodeficiency syndrome (2024 edition), 2024; Chou et al., 2024).

The establishment and maintenance of HIV-1 latency are tightly regulated by the transcriptional state of the viral promoter, the long terminal repeat (LTR), which is highly influenced by host epigenetic mechanisms. Increasing evidence demonstrates that epigenetic regulation plays a central role in controlling HIV-1 transcription by modulating chromatin accessibility, transcription factor recruitment, and RNA processing events, thereby determining whether proviruses remain silent or undergo productive replication (Deeks et al., 2013; Pluta et al., 2020).

Epigenetic regulation refers to reversible mechanisms that modulate gene expression without altering the underlying DNA sequence. Major epigenetic processes include histone modifications, DNA methylation, N^6^-methyladenosine (m^6^A) RNA methylation, and non-coding RNA-mediated regulation (Shin et al., 2026). In the context of HIV-1 infection, these epigenetic processes are dynamically regulated during latency establishment and reversal, involving coordinated actions of chromatin-modifying enzymes, DNA methyltransferases, RNA modification machinery, and regulatory non-coding RNAs. These regulatory events are coordinated by three classes of proteins: writers, which deposit chemical modifications on DNA or histones; erasers, which remove these modifications; and readers, which recognize specific epigenetic marks and recruit downstream effector complexes to regulate transcription (Biswas and Rao, 2018; Honer et al., 2024).

Following integration into the host genome, the proviral DNA is rapidly packaged into chromatin with nucleosomes as the basic unit, each composed of approximately 147 bp of DNA wrapped around a histone octamer containing H2A, H2B, H3, and H4 (Mariño-Ramírez et al., 2005). The integrated HIV-1 provirus utilizes host chromatin regulatory machinery to establish and maintain a transcriptional state, making chromatin remodeling a fundamental determinant of viral latency. Notably, the Nuc-1 nucleosome, which is dynamically regulated by ATP-dependent chromatin remodeling complexes and precisely positioned downstream of the transcription start site (TSS) within the LTR promoter, acts as an important barrier underlying the basal repressive state of the promoter (Dutilleul et al., 2020). Therefore, the establishment of HIV-1 latency is largely governed by the balance between chromatin compaction and accessibility at the LTR region. Its compact arrangement not only impedes transcriptional elongation by RNA polymerase II, but also interferes with the assembly of transcription initiation complexes at the promoter region, thereby restricting LTR transcriptional activity at both the initiation and elongation steps (Dutilleul et al., 2020). These remodeling complexes further coordinate with covalent epigenetic modifications to precisely modulate the chromatin accessibility of the LTR region. On this basis, repressive histone marks (e.g., H3K27me3, H3K9me3) coupled with CpG DNA methylation cooperatively compact chromatin via dense nucleosome packing, limiting access of the transcriptional machinery and enforcing stable proviral silencing (Lange et al., 2020); conversely, activating epigenetic modifications like histone acetylation and crotonylation relax chromatin conformation, expose regulatory binding sites, and enable proviral reactivation upon cellular signals (Van Lint et al., 2013; Jiang et al., 2018). Furthermore, targeted chromatin modifications guided by non-coding RNAs add an additional layer of regulatory precision to the control of viral latency (Zubair et al., 2026). Together, these findings highlight that HIV-1 latency is not a passive consequence of transcriptional inactivity but an actively maintained epigenetic state controlled by multiple interconnected regulatory layers.

Given the central role of epigenetic regulation in controlling HIV-1 transcriptional activity, these mechanisms have become attractive targets for reservoir-directed therapeutic strategies. Approaches including “shock and kill”, which aims to induce proviral expression for immune-mediated clearance, and “block and lock”, which reinforces deep and durable transcriptional silencing, have both incorporated epigenetic interventions as important components (Kessing et al., 2017; Vansant et al., 2020; Kula-Pacurar et al., 2021). However, translating epigenetic interventions into effective HIV cure strategies remains challenging, emphasizing the need for a deeper understanding of epigenetic regulation and rational therapeutic combinations.

Against this background, this review systematically summarizes epigenetic modifications regulating HIV-1 latency, ranging from chromatin remodeling, classic histone, and DNA and m^6^A RNA methylation, dynamic histone acetylation to newly identified regulatory mechanisms including histone crotonylation and non-coding RNA-mediated regulation. We further discuss how these pathways contribute to latency establishment and reversal, evaluate current epigenetic-based therapeutic strategies, including “shock and kill” and “block and lock” approaches, and highlight their limitations, unresolved questions, and future directions. Elucidation of the epigenetic landscape underlying HIV-1 latency will advance mechanistic understanding of proviral silencing and reactivation, and inform the rational design of combinatorial therapeutic regimens toward a functional HIV-1 cure.

## 2 Chromatin remodeling

Nucleosomes, the basic structural units of chromatin, serve as physical barriers to transcription factor binding and RNA polymerase elongation. Their precise positioning at the 5’LTR directly influences the basal transcriptional activity of the virus. Rather than being static, nucleosome positioning is dynamically regulated in coordination with gene expression status. This process, termed ATP-dependent chromatin remodeling, is a conserved structural layer of epigenetic regulation. Driven by ATP hydrolysis, chromatin remodeling complexes (CRCs) represent enzymatically active multi-subunit complexes which employ the energy derived from the hydrolysis of ATP to slide, eject, or exchange nucleosomes along DNA, thereby dynamically adjusting chromatin accessibility at the viral promoter (Reyes et al., 2021).

### 2.1 Nucleosome positioning at the HIV-1 LTR

Following proviral integration into the host DNA, the HIV-1 5’LTR is packaged into nucleosomes arranged at specific intervals but not scattered randomly. This organized nucleosomal pattern was first identified via micrococcal nuclease (MNase) digestion assays in early HIV chromatin studies, which revealed two well-defined nucleosome-protected regions corresponding to Nuc-0 and Nuc-1 across multiple latently infected cell lines (Verdin et al., 1993). Nuc-0 is located upstream of the enhancer region, while Nuc-1 occupies the position downstream from TSS and constitutes an important barrier to the initiation of transcription (Dutilleul et al., 2020).

The stable positioning of Nuc-1 creates a dual barrier to viral gene expression: it physically blocks the formation of the general transcription initiation complex at the core promoter, and prevents the further progression of RNA polymerase II to elongation (Moranguinho and Valente, 2020). In the latent state, this nucleosomal barrier is constantly maintained to keep the LTR in repressive transcriptionally configuration (Jones et al., 2025). Remodeling of Nuc-1 following T-cell activation is tightly connected with the activation of viral gene transcription and confirms its functional importance as a rate-limiting structure checkpoint for LTR activity (Jones et al., 2025). This process is mainly regulated by ATP-dependent chromatin remodeling complexes.

### 2.2 ATP-dependent chromatin remodeling complexes in latency regulation

Depending on their catalytic ATPase domain, these CRCs are classified into four main families: switch/sucrose non-fermenting (SWI/SNF) family, imitation switch (ISWI) family, chromodomain helicase DNA-binding (CHD) family, and the inositol auxotrophy 80 (INO80) family, among which the SWI/SNF family is presently the most thoroughly studied in the context of HIV-1 latency control (Tyagi et al., 2016).

Classical SWI/SNF family includes two antagonistic complexes: BRG1-associated factors (BAF) and polybromo-associated BAF (PBAF). The BAF complex predominates in latently infected T cells and constitutively binds to the LTR promoter. Genetic depletion of its central ATPase subunit BRG1 results in improper positioning of the Nuc-1 and an increase in the basal LTR transcription, suggesting that BAF actively maintains latency by stabilizing the repressive nucleosomal barrier (Rafati et al., 2015). In contrast, the PBAF complex, distinguished by its specific subunit PBRM1, gets recruited to the LTR following the stimulation of the cell by TNF-α treatment or Tat expression. Chromatin immunoprecipitation (ChIP) analysis reveals that PBAF enrichment on the LTR correlates with the ejection of Nuc-1 and the activation of transcription, while deficiency in PBRM1 compromises the Tat-induced transactivation of the promote (Easley et al., 2010).

Recently discovered ncBAF complex, characterized by the presence of its unique subunit, bromodomain-containing protein 9 (BRD9), appears to be another important regulator of HIV-1 latency. Differing from the classical SWI/SNF complexes, ncBAF is characteristic of the HIV-1 LTR in the latent state, where it retains the nucleosomal structure that restricts the transcription through the competition with the viral transactivator Tat for the chromatin docking sites. Inhibition or depletion of ncBAF activity results in the destruction of the repressive chromatin organization and latency reversal, making CRCs potential drug targets for latency interventions (Luk et al., 2025).

Latency homeostasis is also influenced by additional members of the CRC family. For example, Li et al. recently discovered that the INO80-family p400 complex, with EP400 as its core ATPase, acts as a potent restriction factor for HIV transcription. Its subunit DMAP1 competitively binds the basic domain of Tat to block Tat-TAR interaction and repress RNA polymerase II -mediated transcriptional elongation, representing an HIV latency regulatory pathway independent of SWI/SNF complexes (Li C. et al., 2025).

Collectively, the balanced action of activating and repressive CRCs establishes the baseline nucleosomal architecture of the LTR, which works in concert with covalent epigenetic modifications to influence the transcriptional fate of integrated proviruses. Because these complexes also regulate chromatin organization throughout the host genome, however, therapeutic targeting is likely to be highly context dependent and will require strategies that restrict activity to reservoir cells (Reyes et al., 2021; Li C. et al., 2025; Luk et al., 2025).

## 3 Histone, DNA and m^6^A RNA methylation cooperatively establish HIV-1 transcriptional silencing

Among the covalent epigenetic modifications that act in concert with chromatin remodeling, histone methylation, DNA methylation, and m^6^A RNA methylation form a cooperative, multilayered network to establish and maintain HIV-1 transcriptional silencing. At the chromatin level, transcriptional silencing at the HIV-1 5’ LTR is driven by the coordinated, sequential action of histone methylation and DNA methylation (Blazkova et al., 2009; Kauder et al., 2009). In addition to chromatin modifications, m^6^A RNA methylation represents another layer of epitranscriptomic regulation of viral gene expression and interactions with host immune signaling at the post-transcriptional stage.

### 3.1 Histone methylation

Histone methylation is mediated by histone methyltransferases (HMTs), the writers of methylation marks, that catalyze methyl group deposition on lysine and arginine residues of histone tails, thereby regulating chromatin accessibility at the HIV-1 LTR (Persaud and Cates, 2023). Depending on the modified residues and methylation states, histone methylation exerts either repressive such as H3K27me3, H3K9me2/3, and H4K20me1, or activating effects including H3K4me3 on transcription. In latently infected cells, the repressive histone methylation marks prevail at the LTR leading to the establishment of heterochromatin and inhibition of transcriptional activation whereas the activating marks coexist in order to maintain transcriptional poise (Kauder et al., 2009).

Currently, the most well-studied and important repressive pathway of histone methylation involving the polycomb repressive complex 2 (PRC2) and its product H3K27me3 marks is considered the central mechanism regulating HIV-1 latency. As a complex of conserved histone methyltransferases, PRC2 exerts its repressive role predominantly due to the its catalytic subunit, the enhancer of zeste homolog 2 (EZH2), which specifically catalyzes trimethylation of histone H3 lysine 27 (H3K27me3) (Friedman et al., 2011). Depletion of EZH2, or chemical inhibition by the EZH2 inhibitor such as DZNep which reduces global H3K27me3 levels (Sharma et al., 2020), has been demonstrated to reverse HIV-1 latency and promote proviral reactivation (Friedman et al., 2011). Likewise, chemical interference with other core PRC2 members including the EED inhibitors destabilizes PRC2 activity resulting in proviral reactivation (Turner et al., 2020). Additionally, polycomb repressive complex 1 (PRC1) synergizes with PRC2 providing the enhanced epigenetic silencing of the HIV-1 LTR. As one of the important PRC1 components, the chromobox protein homolog 4 (CBX4) enhances enzymatic activity of EZH2 due to the SUMOylation of EZH2, thus reinforcing H3K27me3 methylation (Wu et al., 2022).

In parallel with the PRC-mediated H3K27me3 marks, H3K9 methylation marks represent another important repressive regulatory axis. G9a (EHMT2) and SUV39H1 catalyze H3K9me2 and H3K9me3, respectively, and together with PRC2 components provide increased repression of the HIV-1 LTR with the result of elevated activation threshold of the provirus (Kauder et al., 2009; Nguyen et al., 2017).

In addition to the aforementioned two main repressive pathways, additional HMTs and their associated cofactors provide additional silencing of transcription using complementary ways. For example, the SET and MYND domain-containing protein 2 (SMYD2) promotes H4K20me1 deposition and chromatin compaction by recruitment of repressive protein lethal 3 malignant brain tumor 1 (L3MBTL1) at the LTR (Congdon et al., 2010; Boehm et al., 2017). Importantly, SMYD2 binding at the LTR can be dynamically regulated by inflammatory stimuli such as TNF-α, indicating dynamic regulation by immune signals (Boehm et al., 2017).

Despite the dominance of the repressive marks, the latent HIV-1 LTR is not completely silent, but exhibits a bivalent chromatin state characterized by the simultaneous presence of the repressive mark H3K27me3 and the activating mark H3K4me3 (Nguyen and Karn, 2024). This particular state enables the maintenance of the proviral transcriptional poise and rapid transcriptional activation in case of erasing repressive marks (Nguyen and Karn, 2024). The transition from bivalency to active transcription is driven by histone demethylases, an eraser that remove repressive marks; for instance, the H3K27me3 demethylase UTX/KDM6A erases H3K27me3 marks at the LTR with the result of resolving bivalency and provirus transcriptional activation (Friedman et al., 2011; Nguyen et al., 2021), and pharmacological inhibition of UTX using GSK-J4 provides enhanced repression and supports “block and lock” strategy (Nguyen et al., 2021).

Therapeutically, histone methylation provides entry points for both latency reversal and latency reinforcement. HMT inhibitors such as DZNep, EED inhibitors, and G9a inhibitors have been investigated as latency-reversing approaches by attenuating H3K27me3- or H3K9me2/3-mediated repression at the LTR (Bouchat et al., 2012; Sharma et al., 2020; Turner et al., 2020). Conversely, inhibition of the H3K27me3 demethylase UTX with GSK-J4 supports a “block and lock” direction by maintaining repressive chromatin at the provirus (Nguyen et al., 2021). Future studies should define which reservoir contexts are most dependent on each methyltransferase axis and whether methylation-targeted agents can be combined with immune-based clearance without broad host-chromatin toxicity. Such development will also need to account for functional redundancy among HMTs and demethylases, which may limit the durability of single-pathway interventions and favor combination approaches over monotherapy (Nguyen et al., 2017; Nguyen et al., 2021).

### 3.2 DNA methylation

DNA methylation refers to the addition of methyl groups to cytosine residues within CpG dinucleotides, producing 5-methylcytosine (5mC) through the action of DNA methyltransferases (DNMTs) (Lee et al., 2020). In deeply latent proviruses, CpG islands near the LTR TSS are frequently hypermethylated (Lee et al., 2020), whereas demethylation is associated with transcriptional reactivation (Ishida et al., 2006; Blazkova et al., 2009; Kauder et al., 2009).

Compared with dynamic and reversible histone methylation, DNA methylation acts as a more stable epigenetic layer that reinforces histone-mediated silencing through multi-level crosstalk, rather than initiating silencing independently. As Blazkova et al. demonstrated, transcriptional silencing may be established first before CpG methylation accumulates, suggesting that DNA methylation is a secondary reinforcement mechanism downstream of histone methylation (Blazkova et al., 2009). Heterochromatin protein 1 (HP1) acts as a molecular bridge between histone methylation and DNA methylation that binds the repressive histone mark H3K9me3, and interacts with DNMTs on the other, coordinating deposition of both repressive marks to amplify silencing signals (Smallwood et al., 2007). Moreover, host factors like Apobec3A (apolipoprotein B MRNA editing enzyme catalytic subunit 3A, abbreviated “A3A”) and Piwi (P element-induced Wimpy)-like RNA-mediated gene silencing 4 (PIWIL4) at the HIV-1 LTR assist in the recruitment of HP1 to activate histone methylation via SETDB1 complex, a conserved histone lysine methyltransferase that specifically catalyzes H3K9 trimethylation and drives heterochromatin assembly, further strengthening this synergistic silencing axis (Taura et al., 2019; He et al., 2020). As a parallel repressive pathway, Methylated CpG binding proteins with methyl-CpG-binding domains (MBD), like MBD2, bind to the HIV-1 LTR and facilitate the recruitment of histone deacetylase (HDAC)-containing repressor complexes to silence viral gene expression (Kauder et al., 2009; Shin et al., 2026).

From a therapeutic perspective, DNA methylation is better considered as a reinforcing layer within a broader silencing network rather than as a stand-alone target. Accordingly, therapeutic strategies targeting DNA methylation should take into account its role in maintaining, rather than solely establishing, HIV-1 latency. DNMT inhibition has been reported to partially reverse HIV-1 latency (Bednarik et al., 1987), although its limited single-agent activity indicates that DNA demethylation alone may not be sufficient to overcome the multiple epigenetic barriers maintain proviral silencing (Blazkova et al., 2009; Kauder et al., 2009). Therefore, targeting DNA methylation in combination with other epigenetic approaches may provide a more effective strategy for modulating HIV-1 latency. In the opposite direction, locus-specific reinforcement of DNA methylation could contribute to “block and lock”, but this strategy will require precise delivery to the HIV-1 LTR and careful evaluation of off-target methylation in host genes. These considerations further argue against global DNMT modulation, whose limited specificity and downstream position within the silencing hierarchy are likely to constrain both safety and single-agent efficacy (Bednarik et al., 1987; Blazkova et al., 2009; Kauder et al., 2009).

### 3.3 N^6^-methyladenosine (m^6^A) RNA methylation

Beyond histone and DNA methylation that act at the chromatin level to regulate transcription initiation, m^6^A, abundant internal methylation modification on eukaryotic mRNA, constitutes an epitranscriptomic layer of methylational control that modulates HIV-1 latency at the post-transcriptional stage (Mishra et al., 2024a). The m^6^A modification system comprises three functional classes of regulatory proteins: methyltransferase complexes including METTL3 and METTL14 act as writers that deposit methyl groups on adenosine residues, demethylases such as ALKBH5 and FTO serve as erasers that remove the marks, and a diverse group of m^6^A binding proteins function as readers that influence the fate of modified RNAs by modulating their splicing, nuclear export, stability, and translation efficiency (Mishra et al., 2024b; Ali et al., 2025).

In the process of HIV-1 infection, both viral genomic RNA and host immune-related transcripts undergo m^6^A modification, which collectively shapes the latency state (Mishra et al., 2024a). At the viral level, m^6^A marks are enriched in the coding and untranslated regions of HIV-1 transcripts, where they modulate RNA stability and translational efficiency (Huang et al., 2026). Depletion of the m^6^A writer METTL3 or METTL14 substantially impairs proviral reactivation from latency in CD4^+^ T cell models, whereas enforced expression of METTL3 enhances reactivation competence, indicating that m^6^A deposition on viral RNAs is a permissive factor for productive viral gene expression (Ali et al., 2025). Consistently, pharmacological inhibition of the m^6^A eraser ALKBH5 elevates global m^6^A levels and significantly potentiates the latency-reversing efficacy of classical LRAs such as romidepsin, unveiling a dual-hit strategy that combines chromatin-level epigenetic de-repression with epitranscriptomic enhancement of viral RNA expression.

Therapeutic targeting of m^6^A regulation provides an epitranscriptomic dimension for HIV-1 reservoir intervention. Inhibition of the m^6^A eraser ALKBH5 can increase m^6^A abundance and enhance romidepsin-induced latency reversal, suggesting a potential dual-hit strategy that combines chromatin de-repression with enhanced viral RNA expression. Conversely, targeting m^6^A writers, such as METTL3/METTL14, may reduce proviral reactivation and represents a potential approach for latency stabilization. Recently, pharmacological inhibitors targeting the m^6^A modification machinery have attracted increasing attention. A recent study evaluated the effects of METTL3 inhibitors STM2457, STM3006 and STC-15 on HIV-1 latency regulation and demonstrated that these compounds effectively suppressed HIV-1 latency reversal in both J-Lat cell models and primary CD4^+^ T-cell latency models, highlighting the potential of m^6^A-targeting strategies to limit viral reactivation (Mishra et al., 2026). Among these inhibitors, STC-15 has advanced into phase I and II clinical trials (Mishra et al., 2026); however, its therapeutic potential in HIV-1 infection and possible cytotoxic effects require further investigation. Interpretation of these approaches is complicated by the bidirectional and transcript-specific effects of m6A: systemic perturbation may also alter host immune RNA programs, and whether writer or eraser inhibition offers the more useful therapeutic direction remains unresolved (Mishra et al., 2024a; Mishra et al., 2024b; Ali et al., 2025; Mishra et al., 2026).

Collectively, m^6^A RNA methylation extends the methylational regulatory network of HIV-1 latency from the chromatin to the post-transcriptional level. Together with histone and DNA methylation, it constitutes a multi-layered methylation regulatory network that offers expanded druggable targets for reservoir intervention.

## 4 Histone acetylation and HDAC-dependent regulation of HIV-1 transcription

Histone acetylation also participates in the regulation of HIV-1 transcription; however, unlike the aforementioned methylation, which establishes and maintains latency to repress transcription, acetylation acts primarily by increasing chromatin accessibility at the LTR, thereby promoting transcriptional activation. This regulatory system works under the control of three types of participants: histone acetyltransferases (HATs) that act as “writers” (Yang, 2004), HDACs that are “erasers”, and acetyl-lysine binding proteins as “readers”, which together affect the transcriptional state of the latent provirus.

### 4.1 Histone acetylation and chromatin accessibility

An important early event of HIV-1 reactivation is the formation of histone acetylation at the LTR, specifically acetylation of histone H3 lysine 27 (H3K27ac) and acetylation of histone H3 lysine 9 (H3K9ac) that are linked to the switch from transcriptionally restrictive to transcriptionally competent chromatin (Mbonye and Karn, 2014). These two modifications are catalyzed by distinct subfamilies of HATs, the enzymatic addition of acetyl groups to lysine residues on histone tails, and correspondingly regulate different steps of transcriptional activation, collectively driving the viral promoter from a silent to an active state (Legube and Trouche, 2003).

Specifically, the transcriptional coactivators p300 and CREB-binding protein (CBP) are the primary enzymes responsible for H3K27ac deposition at the LTR (Horvath and Sadowski, 2024). They deposit acetyl groups on H3K27 residues within nucleosome-dense regions downstream of the LTR, particularly the Nuc-1 region that constitutes a transcriptional barrier. By neutralizing the positive charge of histones and disrupting electrostatic interactions between histones and DNA, they relax the chromatin and relieve the physical block imposed by nucleosomes on the transcriptional machinery (Wang et al., 2008; Tie et al., 2009; Stasevich et al., 2014; Raisner et al., 2018; Zhou et al., 2019). For example, Zhang et al. reported that acetylation of H3K27 increased substantially at the Nuc-1 region upon reactivation of proviruses within Jurkat T cells, which reflects the significance of p300/CBP-mediated acetylation for remodeling nucleosomal barriers at the LTR (Zhang et al., 2017).

Notably, H3K27 acetylation and H3K27 trimethylation are mutually exclusive at the same lysine residue, forming a direct molecular switch between silencing and activation at the LTR (Tie et al., 2009). This mechanistic relationship underpins the therapeutic synergy between HDAC inhibitors (HDACi) and EZH2 inhibitors that combined targeting of the erasers for both marks can cooperatively relieve dual repression at this locus and achieve more robust latency reversal, a combination strategy validated in multiple preclinical studies (Tripathy et al., 2015; Maina et al., 2021). While direct HAT activators could theoretically enhance acetyl mark deposition, they have not advanced into clinical development for HIV latency due to poor genome-wide specificity (Wang et al., 2009; Almolhim, 2026).

Meanwhile, other HAT family members, including PCAF and GCN5, predominantly mediate H3K9ac modification, with functions focused on the regulation of transcription initiation and elongation (Horvath and Sadowski, 2024). H3K9ac is mainly enriched at the Nuc-0 region near the TSS, and accumulates at early stage of reactivation prior to viral mRNA synthesis. On one hand, it participates in the assembly of transcription initiation complexes; on the other hand, it recruits the super elongation complex (SEC), driving the transition of RNA polymerase II from promoter-proximal pausing to productive elongation and enabling subsequent full-length viral transcription. Therapeutically, this step represents a critical node where acetylation signals are transduced into downstream transcriptional output, and it is the primary functional target of BET family reader proteins (Alamer et al., 2021). By modulating the reading efficiency of acetyl marks at this site, viral transcription can be bidirectionally tuned without altering global acetylation deposition levels, which forms the mechanistic basis for BRD4-targeted pharmacological interventions (Boehm et al., 2013; Conrad et al., 2017; Alamer et al., 2021; Turner et al., 2025).

Acetylation modifications mediated by these two classes of HATs act synergistically that H3K27ac driven by p300/CBP is responsible for opening the chromatin barrier, whereas H3K9ac mediated by PCAF/GCN5 bridges transcription initiation and elongation to propel the transcriptional process. Together, they operate coordinately that rapidly primes LTR chromatin for transcription and facilitates proviral reactivation.

### 4.2 HDAC networks

The transcriptional activation effect mediated by histone acetylation is not an unrestricted process, but is maintained in dynamic homeostasis together with deacetylation, with HDACs serving as an important counter-regulatory factors. As epigenetic “erasers”, HDACs remove acetyl groups from histone tails, counteract the chromatin-opening effect of HATs, and constitutively maintain the basal repressive chromatin state at the LTR region (Legube and Trouche, 2003). HDAC1, HDAC2 and HDAC3 are the core isoforms enriched at the HIV-1 LTR, recruited via adaptor proteins such as YY1 and FKBP3 to form a multilayered repressive network (Yang et al., 2021).

A typical feature of this network is functionally complementary among HDAC family members that inhibition of a single HDAC isoform is insufficient to induce robust histone acetylation, as remaining isoforms can compensate for the lost function (Hess et al., 2022). In 2023, Peter et al. further corroborated this notion, demonstrating that targeting HDAC1/2 or HDAC3 alone is sufficient to block latency establishment, whereas simultaneous inhibition of all three HDAC enzymes is required for latency reversal (Peterson et al., 2023). This isoform compensation directly explains the limited single-agent efficacy of HDACi in clinical settings, and guides the selection of drug candidates and combination regimen design.

Although HDACi such as panobinostat, vorinostat (SAHA) and romidepsin have been confirmed to effectively elevate histone acetylation levels and induce low-level HIV-1 transcription *in vivo*, validating the feasibility of targeting HDACs for latency intervention, the complexity of the HDAC network poses obstacles to achieving robust latency reversal and reservoir reduction with single HDACi (Elliott et al., 2014; Rasmussen et al., 2014; Søgaard et al., 2015).

Pan-HDACi were the first class of latency-reversing agents (LRAs) tested in clinical trials. Vorinostat (SAHA) was the first HDACi evaluated in clinical studies and was shown to induce robust histone acetylation and viral reactivation in latently infected cell lines and primary resting CD4^+^ T cells *ex vivo* (Archin et al., 2012). Panobinostat, another pan-HDAC inhibitor, has been evaluated in phase I/II clinical trials; when administered with ART, it increases cell-associated HIV-1 RNA levels (Rasmussen et al., 2014). Romidepsin, a class I-selective HDAC inhibitor, has also shown strong latency-reversing activity *ex vivo* and *in vivo* (Søgaard et al., 2015; McMahon et al., 2021). However, broad-spectrum HDAC inhibition carries significant off-target toxicity due to genome-wide effects on host gene expression. Importantly, accumulating evidence demonstrates dose-dependent immunotoxicity of these agents on effector immune cells: romidepsin and panobinostat have been shown to impair CD8^+^ T cell proliferation, cytokine secretion, degranulation capacity and cytolytic function, which may compromise the “kill” phase of the strategy even as they successfully induce viral transcription, representing a core translational bottleneck for this drug class (Jones et al., 2014; Zhao et al., 2019; Rosás-Umbert et al., 2020).

Given the complexity of the HDAC network, pan-HDACi or multi-target combination regimens may offer a means to overcome current therapeutic bottlenecks. For example, the recent identification of the new subtype-selective HDACi, CC-4a, is characterized by low toxicity and effective latency reversal by promoting the NF-κB signaling pathway activation as well as exhibiting synergy with the protein kinase C agonist prostratin (Lu et al., 2021).

Subtype-selective HDACi with improved safety profiles have emerged as promising next-generation LRAs. Chidamide is an oral subtype-selective HDAC inhibitor that preferentially targets class I HDACs (e.g., HDAC1, HDAC2 and HDAC3), as well as the class IIb isoform HDAC10 (Ning et al., 2012). It has an established clinical safety profile in oncology and exhibits more favorable pharmacokinetic properties and lower systemic cytotoxicity than broad-spectrum pan-HDACi (Ning et al., 2012). Subsequent preclinical data confirm that chidamide elevates histone acetylation levels at the HIV-1 LTR and effectively induces proviral reactivation in primary CD4^+^ T cells from ART-suppressed people living with HIV (PLWH) (Kuai et al., 2018). In a recently published phase II single-arm clinical study, the combination of oral chidamide and the anti-PD-L1 antibody ASC22 achieved significant latent reservoir activation in virologically suppressed PLWH, with mild adverse events and good overall tolerability (Wu et al., 2024). Moreover, a measurable reduction in reservoir size was observed in a subset of participants with restored HIV-specific CD8^+^ T cell function, suggesting a potential step toward the clinical translation of chidamide-based reservoir-targeting strategies (Wu et al., 2024). Despite improved safety, subtype-selective inhibitors remain constrained by HDAC family compensation, with weaker single-agent latency-reversing potency than pan-HDACi, and typically require combination with agents targeting other epigenetic pathways to achieve optimal effects.

Accordingly, HDACi should be discussed not only as a potential therapeutic class, but also as direct functional probes of the histone-acetylation mechanism. Vorinostat, panobinostat, romidepsin, and chidamide have shown capacity to increase histone acetylation and induce HIV transcription (Archin et al., 2012; Ning et al., 2012; Elliott et al., 2014; Rasmussen et al., 2014; Søgaard et al., 2015; Kuai et al., 2018; McMahon et al., 2021; Wu et al., 2024), yet clinical reservoir reduction remains inconsistent. This discrepancy arises largely from the substantial gap between preclinical cell-line models and clinical outcomes: HDACi consistently achieve strong reactivation in transformed cell lines such as J-Lat, but show highly variable and often modest effects in primary resting CD4^+^ T cells and *in vivo* (Wightman et al., 2013; Almolhim, 2026), driven by reservoir heterogeneity, tissue drug penetration barriers, and the more compact chromatin state of authentic latent reservoirs (Gallego-Cortés et al., 2025; Ji et al., 2025; Almolhim, 2026). A key future direction is to pair HDACi with agents that improve immune clearance or target complementary epigenetic barriers, while monitoring potential effects on CD4^+^ and CD8^+^ T-cell function. Together, these observations indicate that future HDACi regimens will need to couple transcriptional induction with reservoir clearance while minimizing toxicity, preserving immune-effector function, and accounting for compensatory HDAC activity (Archin et al., 2012; Elliott et al., 2014; Jones et al., 2014; Rasmussen et al., 2014; Søgaard et al., 2015; Zhao et al., 2019; Rosás-Umbert et al., 2020; McMahon et al., 2021; Peterson et al., 2023).

### 4.3 BRD4-mediated transcriptional elongation

The biological effects of histone acetylation are transferred by the reader proteins recognizing acetyl-lysine marks and translating the epigenetic signal into the transcriptional outputs. Currently, one of the most extensively investigated acetyl-lysine readers in the regulation of HIV-1 transcription is BRD4, which belongs to the bromodomain and extra-terminal (BET) family.

Accumulating evidence showed that BRD4 exerts context-dependent dual regulatory effects on viral transcription. The long isoform of BRD4 (BRD4-L) recognizes acetylated histones using two bromodomains (BD1 and BD2) and recruits the positive transcription elongation factor b (P-TEFb) via its ET domain. The interaction releases RNA polymerase II from promoter-proximal pausing and stimulates productive transcriptional elongation, thereby linking histone acetylation to active viral transcription (Zeng and Zhou, 2002; Jang et al., 2005; Mujtaba et al., 2007; Conrad et al., 2017).

This activating function of BRD4-L can be pharmacologically exploited for “block and lock” strategies. BD1 domain-selective inhibitors such as ZL0580 primarily target the BRD4-L isoform: by selectively binding to the BD1 domain of BRD4, they block chromatin association and P-TEFb recruitment of the long isoform at the LTR, attenuate its transcriptional activating effect, and ultimately deepen viral transcriptional silencing (Alamer et al., 2021). In cell models and primary PBMCs from people living with HIV, ZL0580 significantly suppresses viral transcription and delays viral rebound after treatment interruption; when combined with LEDGF/p75 inhibitors (LEDGINs), it further reinforces the “block and lock” effect via complementary silencing at the integration site and transcriptional levels (Pellaers et al., 2025). A key unresolved question for this strategy is whether drug-induced silencing can be durably maintained after drug withdrawal, or whether it merely delays rather than prevents viral rebound.

In contrast, the short isoform of BRD4 (BRD4-S) s involved in the maintenance of latency because it competes with HIV-1 Tat protein for P-TEFb binding, and recruits SWI/SNF chromatin remodeling complexes to preserve the transcriptionally restrictive nucleosomal architecture at the LTR. Correspondingly, targeting the repressive BRD4-S isoform supports a “shock and kill” therapeutic direction. Pan-BET inhibitors including JQ1 non-selectively block the binding of BRD4 to acetylated chromatin. In latently infected cells, their predominant net effect is to displace the repressive BRD4-S from the LTR region, relieving isoform-mediated transcriptional inhibition and triggering reactivation of latent proviruses (Filippakopoulos et al., 2010; Darcis et al., 2015; Laird et al., 2015). Because simultaneous targeting of the eraser and reader layers of the acetylation axis produces synergistic effects, JQ1 exerts a well-documented synergistic effect with the HDACi SAHA in latency reversal (Filippakopoulos et al., 2010; Darcis et al., 2015; Laird et al., 2015). However, pan-BET inhibitors carry substantial risk of genome-wide off-target effects, as BRD4 is a ubiquitous regulator of host gene transcription; broad inhibition can widely perturb the host transcriptome, leading to immune dysregulation and systemic toxicity, which remains a major barrier to clinical translation (Divakaran et al., 2023; Turner et al., 2025).

This dual function has likewise been validated through pharmacological approaches. BD1 domain-selective inhibitors such as ZL0580 primarily target the BRD4-L. By selectively binding to the BD1 domain of BRD4, they block chromatin association and P-TEFb recruitment of the long isoform at the LTR, attenuate its transcriptional activating effect, and ultimately deepen viral transcriptional silencing (Alamer et al., 2021). In cell models and primary PBMCs from people living with HIV, ZL0580 significantly suppresses viral transcription and delays viral rebound after treatment interruption; when combined with LEDGF/p75 inhibitors (LEDGINs), it further reinforces the “block and lock” effect (Pellaers et al., 2025). Conversely, Pan-BET inhibitors including JQ1 non-selectively block the binding of BRD4 to acetylated chromatin. In latently infected cells, their predominant effect is to displace the repressive BRD4-S from the LTR region, relieving isoform-mediated transcriptional inhibition and triggering reactivation of latent proviruses and exerts a synergistic effect with the HDACi SAHA (Filippakopoulos et al., 2010; Darcis et al., 2015; Laird et al., 2015).

Beyond classical BET family acetyl-lysine reader proteins, BRD9 has recently been identified as a novel non-BET epigenetic reader that links histone acetylation signals to chromatin remodeling in HIV-1 latency control (Luk et al., 2025). It specifically recognizes acetylated histone modifications at the LTR via its intrinsic bromodomain, accumulates at viral regulatory regions to recruit the ncBAF, ultimately maintaining viral latency by competing with the Tat protein for binding sites and inhibiting transcriptional elongation (Luk et al., 2025). Furthermore, Tsz-Yat Luk et al. demonstrated that disruption of BRD9 reader function via genetic knockdown, pharmacological inhibition or proteolysis-targeting chimeras (PROTAC) -mediated targeted degradation effectively reverses HIV-1 latency in T cell lines, primary resting memory CD4^+^ T cells, and PBMCs from people living with HIV. Moreover, combined inhibition of BRD9 and BRD4 simultaneously abrogates the function of two classes of acetyl-lysine readers, resulting in more robust viral reactivation (Luk et al., 2025). BRD9-targeted approaches currently remain at the preclinical stage, and *in vivo* delivery efficiency and off-target risks remain to be further validated (Luk et al., 2025).

The acetyl-lysine reader pathway also illustrates how the same epigenetic mechanism can support opposite therapeutic goals. Pan-BETi such as JQ1 can act as LRAs by relieving BRD4-mediated repression and synergizing with HDACi (Filippakopoulos et al., 2010; Zhu et al., 2012; Boehm et al., 2013; Darcis et al., 2015; Laird et al., 2015), whereas BD1-selective BRD4 modulation with ZL0580, especially in combination with LEDGINs, reinforces transcriptional silencing and delays rebound (Alamer et al., 2021; Pellaers et al., 2025). BRD9 inhibition or degradation represents a newer reactivation strategy (Luk et al., 2025). Collectively, the development of isoform-selective BET modulators and targeted protein degraders represents a key direction to improve target specificity and reduce off-target effects, but further validation in primary reservoir models and *in vivo* systems is required. Future studies should compare BET- and BRD9-targeted approaches across primary reservoir models and define how to minimize broad perturbation of host transcription. This need for selectivity is particularly important because BET-family readers participate broadly in host transcription, whereas BRD9-directed interventions have not yet progressed beyond preclinical evaluation (Alamer et al., 2021; Divakaran et al., 2023; Luk et al., 2025; Turner et al., 2025).

## 5 Multidimensional expansion of the HIV-1 regulatory network

In addition to classic histone and DNA modifications (**Figure 1**), emerging evidence indicates that additional epigenetic regulatory dimensions further modulate proviral transcription.

**Figure 1:**
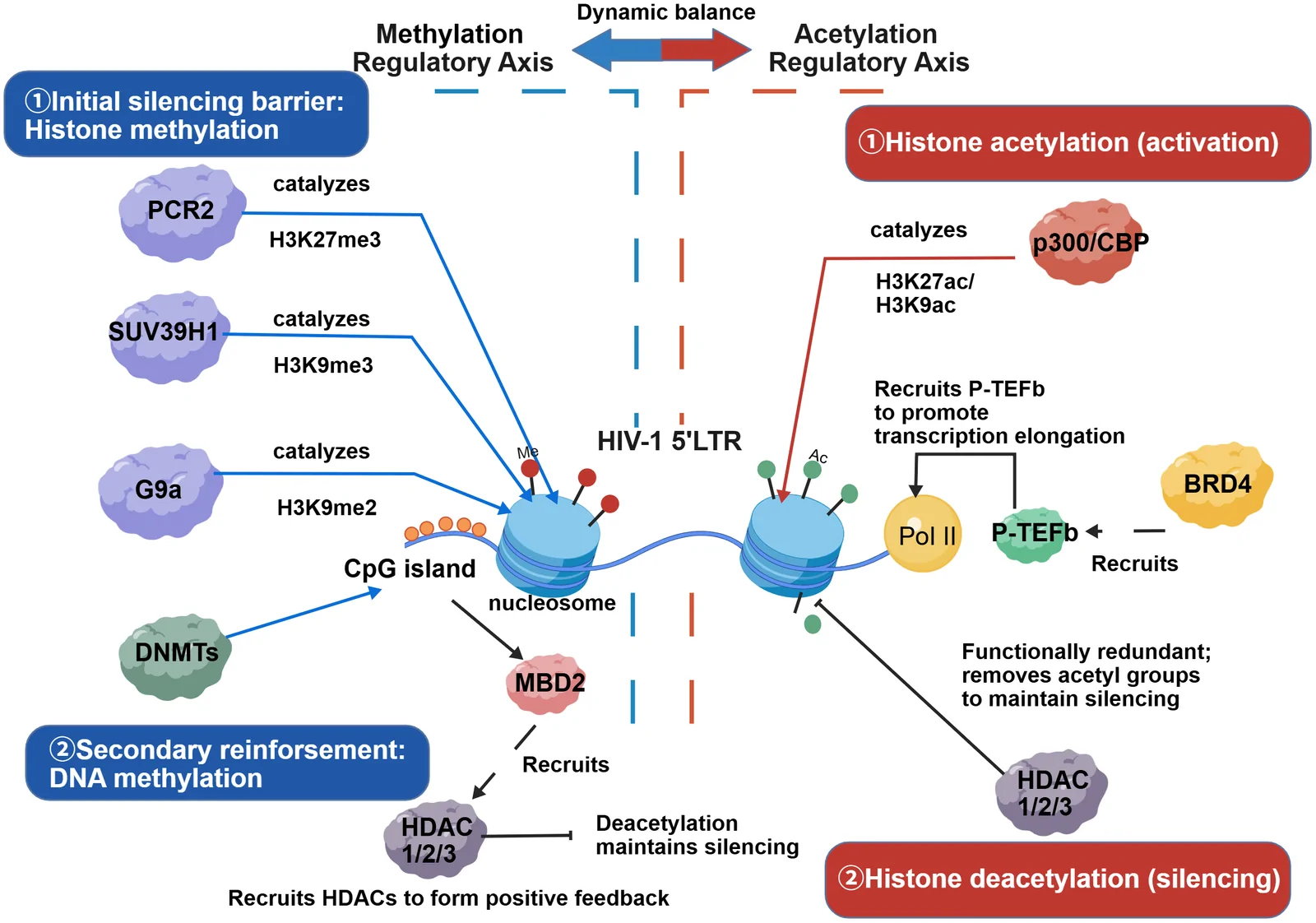
Classical epigenetic regulatory network controlling HIV-1 5’ LTR transcriptional state. Left panel: The blue methylation regulatory axis establishes epigenetic silencing in a layered two-step process, with blue arrows indicating catalytic or recruitment effects. Initial silencing barrier: Histone methyltransferases including PRC2, SUV39H1 and G9a deposit repressive methylation marks (Me) on nucleosomes at the 5’ LTR, directly compacting chromatin structure. Secondary reinforcement: DNMTs mediate methylation of the LTR CpG island; methylated CpGs are recognized by MBD2, which further recruits HDAC1/2/3 to form a self-sustaining positive feedback loop and chemically lock the silent state. Right panel: The red acetylation regulatory axis controls transcriptional activation, with red arrows indicating catalytic effects and black arrows indicating recruitment effects. Histone acetyltransferases p300/CBP deposit activating acetylation marks (Ac) to loosen the chromatin which in turn facilitates BRD4-mediated recruitment of P-TEFb for RNA polymerase II elongation transcription. HDAC1, HDAC2 and HDAC3 remove acetyl groups from histone lysine residues to promote transcriptional silencing. An important two-headed blue-red arrow highlights the dynamic balance between these two opposing axes, determining whether the integrated provirus will remain latent or be reactivated. This figure is generated using Generic Diagramming Platform (GDP, https://BioGDP.com) (Jiang et al., 2025).

### 5.1 Histone crotonylation

Histone crotonylation (Kcr), a metabolically sensitive chromatin modification that links metabolism to the transcriptional competence of HIV-1 proviruses, was first identified in 2011 by Zhao et al. through high-resolution mass spectrometry-based proteomic profiling (Tan et al., 2011). As a novel and evolutionarily conserved histone acyl modification, histone crotonylation is highly enriched at the promoters and enhancers of actively transcribed genes (Tan et al., 2011). Similar to acetylation, crotonylation represents a long fatty acyl chain with the presence of a double carbon bond that allows greater transcriptional potency (Tan et al., 2011). The deposition of Kcr in the chromatin is carried out by p300/CBP that have both activities of acetylation and crotonylation. As a result, the ability to write different acyl modifications on lysine residues provides plasticity within this family of transcriptional coactivators (Tan et al., 2011; Mori and Valente, 2020). Moreover, the production of crotonyl-CoA, the metabolic donor for Kcr modification, is regulated by enzymes like acyl-CoA synthetase short-chain family member 2 (ACSS2). This enzyme thus links cellular metabolism directly to chromatin modifications. In HIV-1 latency models, modulation of ACSS2 expression alters crotonylation levels at the LTR such that ACSS2 upregulation results in increased H3 crotonylation and proviral reactivation, while ACSS2 inhibition reduced crotonylation and impaired reactivation (Jiang et al., 2018). These findings show that the regulation of metabolism can influence the formation of epigenetic marks that regulate proviral transcription.

In addition to metabolic control, histone crotonylation is also regulated by chromatin-associated enzymatic activities. For instance, HDAC3 functions as a histone decrotonylase, and its activity is linked to Kcr turnover (Li et al., 2026). Pharmacological inhibition of HDAC3, such as with citarinostat, increases histone crotonylation levels and facilitates latency reversal, highlighting crotonylation as a therapeutically targetable epigenetic modification (Li et al., 2026).

Consistent with its transcription-activating function, crotonylation-targeted strategies fall primarily within the “shock and kill” therapeutic framework. Potential approaches include increasing ACSS2-dependent crotonyl-CoA production to promote LTR accessibility or inhibiting HDAC3-mediated decrotonylation to stabilize activating crotonyl marks (Jiang et al., 2018; Li et al., 2026). However, because crotonylation is closely linked to cellular metabolism and widely distributed across the host genome, global manipulation may broadly rewire metabolic gene expression in both reservoir and immune effector cells, raising off-target toxicity concerns (Ji et al., 2025). Notably, current mechanistic evidence is derived mostly from transformed T-cell lines, and whether Kcr exerts consistent regulatory effects in primary resting CD4^+^ T cells and myeloid reservoirs remain to be fully validated (Jiang et al., 2018). Accordingly, translation of this approach will depend on whether proviral crotonylation can be modulated without broadly perturbing metabolic and transcriptional programs in reservoir and immune-effector cells (Jiang et al., 2018; Ji et al., 2025; Li et al., 2026).

### 5.2 Non-coding RNA-mediated regulation

At the sequence-guided regulatory level, non-coding RNAs play locus-specific role by recruiting chromatin-modifying complexes to defined genomic regions. The cellular long non-coding RNAs (lncRNAs) exhibit strong cell type specificity in regulating the proviral transcriptional state (Crespo et al., 2022; Yuan et al., 2023).

The host lncRNA MALAT1 promotes transcriptional activation by binding PRC2 and sequestering it away from the HIV-1 LTR, resulting in reduced H3K27me3 deposition and alleviation of transcriptional repression (Qu et al., 2019). In contrast, the viral antisense RNA (ASP RNA) functions as a viral guide RNA, directing PRC2-mediated H3K27me3 at the HIV-1 LTR region (Zapata et al., 2017).

These opposing RNA-mediated mechanisms suggest that both host and viral transcripts regulate the proviral transcriptional state via their distinct interaction mechanisms.

Notably, the capability of RNA to recruit chromatin modification proteins to specific loci is useful for the design of RNA-based therapies using synthetic RNAs for targeted manipulation of the latency status of HIV-1 proviruses. Two well-validated synthetic RNA strategies include promoter-targeted siRNAs that establish durable transcriptional silencing via heterochromatin induction (Ahlenstiel et al., 2015), and engineered antisense transcript mimics that recruit repressive epigenetic complexes such as PRC2 to the viral 5’LTR region (Li R. et al., 2025). These RNA-guided approaches are most naturally aligned with “block and lock” because they can recruit repressive chromatin-modifying complexes to the viral promoter in a sequence-guided manner (Ahlenstiel et al., 2015; Li R. et al., 2025). In parallel, targeting host repressive ncRNAs or augmenting MALAT1 function represents an emerging “shock and kill” route to indirectly displace PRC2 from the LTR (Qu et al., 2019), though this direction remains largely conceptual due to broad genomic targets of host lncRNAs (Boliar and Russell, 2021).

Despite the advantage of locus specificity over global small-molecule epigenetic drugs (Morris, 2008), clinical translation of RNA-guided epigenetic therapies faces multiple barriers. First, efficient and durable delivery to long-lived reservoir cells, particularly tissue-resident memory T cells and myeloid cells, remains a major technical challenge (Zubair et al., 2026). Second, exogenous RNA molecules may trigger innate immune activation via pattern recognition receptors, causing non-specific inflammation and cytotoxicity (Karikó et al., 2005). Third, the efficacy of ncRNA-guided chromatin modification may vary across different HIV-1 integration sites, as proviral chromatin state is strongly influenced by flanking host genomic sequences (Jordan et al., 2003). Finally, whether RNA-induced epigenetic silencing can be stably maintained long-term without continuous dosing remains unresolved, with current data limited to short-term *in vitro* and *ex vivo* models (Tolomeo and Cascio, 2026). Taken together, these issues make delivery, immune compatibility, and consistency across integration sites central considerations in determining whether the locus specificity observed experimentally can be retained *in vivo* (Jordan et al., 2003; Karikó et al., 2005; Morris, 2008; Boliar and Russell, 2021; Tolomeo and Cascio, 2026; Zubair et al., 2026).

Taken together, beyond classical histone and DNA epigenetic modifications, metabolic-dependent histone crotonylation and non-coding RNA-based regulatory pathways further expand the multilayered HIV-1 latency regulatory network. Histone crotonylation bridges cellular metabolism and viral transcription, with ACSS2 and HDAC3 dynamically modulating LTR crotonylation levels to control proviral reactivation. Moreover, host and viral non-coding RNAs exert opposite regulatory effects on HIV-1 transcription via targeted recruitment of CRCs. Collectively, these two novel regulatory dimensions enrich the epigenetic landscape of HIV-1 latency and provide promising alternative targets for future latency-targeted therapeutic strategies.

## 6 Discussion

The studies reviewed here suggest that HIV-1 latency is regulated by several coordinated epigenetic mechanisms. At the chromatin level, ATP-dependent chromatin remodeling complexes influence nucleosome positioning at the LTR, thereby affecting the transcriptional accessibility of the viral promoter. Histone methylation and DNA methylation can act together to promote and maintain proviral silencing, whereas changes in histone acetylation and crotonylation are associated with chromatin opening and transcriptional reactivation in response to cellular stimuli. In addition to these chromatin-based mechanisms, m^6^A RNA methylation and non-coding RNAs provide further levels of regulation, linking transcriptional control at the LTR with post-transcriptional regulation of viral gene expression (**Table 1**).

**Table 1:** Epigenetic regulatory layers in HIV-1 latency: functional roles, therapeutic opportunities, and limitations.

Epigenetic modification / regulatory layer	Role in HIV-1 latency	Associated therapeutic approaches	Key limitations
Chromatin remodeling and nucleosome positioning	Nuc-1 positioning creates a transcriptional barrier at the LTR; BAF, ncBAF/BRD9 and p400-related complexes help maintain restrictive chromatin, whereas PBAF supports Tat-driven activation (Verdin et al., 1993; Easley et al., 2010; Rafati et al., 2011; Li C. et al., 2025; Luk et al., 2025).	Targeting chromatin remodelers or acetyl-lysine reader-associated remodeling complexes, including BRD9 inhibition/degradation, may promote latency reversal; targeted chromatin editing may support locus-specific control (Li C. et al., 2025; Luk et al., 2025).	Chromatin remodelers have broad host-genome functions; effects are context- and cell-type-dependent, and selective delivery to reservoir cells remains difficult.
Histone methylation	Repressive H3K27me3, H3K9me2/3 and H4K20me1 marks compact LTR chromatin and raise the activation threshold, while bivalent H3K27me3/H3K4me3 preserves transcriptional poise (Congdon et al., 2010; Friedman et al., 2011; Boehm et al., 2017; Nguyen et al., 2017; Sharma et al., 2020; Turner et al., 2020; Nguyen et al., 2021; Wu et al., 2022; Nguyen and Karn, 2024).	EZH2/DZNep, EED inhibitors and G9a inhibitors have been explored for latency reversal; UTX inhibition by GSK-J4 may reinforce silencing in a block-and-lock direction (Bouchat et al., 2012; Sharma et al., 2020; Turner et al., 2020; Nguyen et al., 2021).	Multiple HMTs and demethylases act redundantly; broad modulation may affect host chromatin and may not produce durable reservoir control as monotherapy.
DNA methylation	CpG methylation at the LTR provides a stable downstream lock that cooperates with HP1, DNMTs, MBD2 and HDAC-containing repressor complexes to reinforce silencing (Bednarik et al., 1987; Ishida et al., 2006; Smallwood et al., 2007; Blazkova et al., 2009; Kauder et al., 2009; Taura et al., 2019; He et al., 2020; Lee et al., 2020).	DNMT inhibition can partially reverse latency; locus-specific DNA methylation or demethylation could theoretically support block-and-lock or shock-and-kill strategies (Bednarik et al., 1987; Blazkova et al., 2009; Kauder et al., 2009).	Global DNMT targeting is non-specific and potentially toxic; DNA methylation is often secondary to histone-mediated silencing, limiting single-agent efficacy.
m^6^A RNA methylation	m^6^A regulates viral and host RNA stability, export and translation, and METTL3/METTL14-dependent m^6^A deposition supports productive reactivation competence (Mishra et al., 2024a; Mishra et al., 2024b; Ali et al., 2025; Huang et al., 2025).	ALKBH5 inhibition can potentiate romidepsin-mediated latency reversal, whereas METTL3 inhibitors may attenuate latency reversal and could be explored for latency-stabilizing strategies (Yankova et al., 2021; Fiorentino et al., 2023; Guirguis et al., 2023; Harrahill and Hadden, 2024; Mishra et al., 2024a; Mishra et al., 2024b; Ali et al., 2025; Huang et al., 2025; Mishra et al., 2026).	mA has bidirectional and transcript-specific effects; systemic modulation may disturb host immune RNA programs, and optimal therapeutic direction remains unresolved.
Histone acetylation and HDAC-dependent regulation	H3K27ac and H3K9ac increase LTR accessibility and transcriptional initiation/elongation, whereas HDAC1/2/3 maintain a deacetylated repressive state (Legube and Trouche, 2003; Wang et al., 2008; Tie et al., 2009; Mbonye and Karn, 2014; Stasevich et al., 2014; Gates et al., 2017; Zhang et al., 2017; Raisner et al., 2018; Zhou et al., 2019; Yang et al., 2021; Hess et al., 2022; Peterson et al., 2023; Horvath and Sadowski, 2024).	HDACi including vorinostat, panobinostat, romidepsin, chidamide and CC-4a can induce HIV transcription and are central LRAs in shock-and-kill strategies (Archin et al., 2012; Ning et al., 2012; Elliott et al., 2014; Rasmussen et al., 2014; Søgaard et al., 2015; Kuai et al., 2018; Lu et al., 2021; McMahon et al., 2021; Wu et al., 2024).	Single HDACi often induces transcription without consistent reservoir reduction; toxicity, immune-effector impairment and compensatory HDAC networks constrain translation.
BET/BRD4 and BRD9 acetyl-lysine reader pathways	BRD4 isoforms link acetylated chromatin to P-TEFb-dependent elongation or repression; BRD9 recruits ncBAF to maintain latency (Zeng and Zhou, 2002; Jang et al., 2005; Bisgrove et al., 2007; Mujtaba et al., 2007; Conrad et al., 2017; Mbonye et al., 2018; Luk et al., 2025).	Pan-BET inhibitors such as JQ1 reactivate latency and synergize with HDACi, whereas ZL0580 plus LEDGINs reinforces block-and-lock silencing; BRD9 inhibitors/degraders promote reactivation (Filippakopoulos et al., 2010; Zhu et al., 2012; Boehm et al., 2013; Darcis et al., 2015; Laird et al., 2015; Alamer et al., 2021; Luk et al., 2025; Pellaers et al., 2025).	Reader proteins regulate many host transcriptional programs; pan-BET inhibition lacks locus specificity, while BRD9-targeted approaches remain largely preclinical.
Histone crotonylation	Crotonylation links cellular metabolism to LTR accessibility; ACSS2-dependent crotonyl-CoA production and HDAC3-mediated decrotonylation modulate proviral reactivation (Tan et al., 2011; Jiang et al., 2018; Mori and Valente, 2020; Li et al., 2026).	ACSS2 modulation or HDAC3 inhibition, such as citarinostat-mediated decrotonylation blockade, may facilitate latency reversal (Jiang et al., 2018; Li et al., 2026).	Because crotonylation is metabolism-sensitive, therapeutic manipulation may broadly rewire host metabolic and transcriptional programs.
Non-coding RNA-mediated regulation	Host and viral non-coding RNAs recruit or displace chromatin-modifying complexes at the LTR; MALAT1 relieves PRC2-mediated repression, whereas ASP RNA promotes PRC2 recruitment (Zapata et al., 2017; Qu et al., 2019; Crespo et al., 2022; Yuan et al., 2023).	Promoter-targeted siRNAs and engineered antisense transcript mimics can guide heterochromatin formation and support block-and-lock strategies (Ahlenstiel et al., 2015; Li R. et al., 2025).	Efficient delivery, innate immune activation, off-target RNA effects and integration-site-dependent specificity remain major barriers.

Although considerable progress has been made in understanding the epigenetic regulation of HIV-1 latency and in testing related therapeutic approaches, several challenges still limit their clinical application. “Shock and kill” strategy, aimed at reversing latency, have shown the ability to induce transcription but are limited by the robustness of overlapping repressive mechanisms, reservoir heterogeneity, and potential off-target effects on host gene expression (Addissouky, 2025). In contrast, “block and lock” strategy seeks to maintain proviral silencing and reduce the risk of viral rebound, but its application is limited by challenges in delivery, the long-term stability of epigenetic repression, and variable responses among provirus integrated at different genomic sites (Tolomeo and Cascio, 2026) (**Figure 2**, **Table 2**). These barriers suggest that epigenetic approaches may be insufficient for either latency reversal or sustained viral silencing. Therefore, combination strategies that act on multiple epigenetic pathways may be worth further consideration. Similar ideas have been explored in cancer research, where combined targeting of different epigenetic regulators has shown stronger effects than single-agent treatment in some settings. For example, HDACi combined with BET inhibitors have shown synergistic antitumor effects in preclinical models of multiple myeloma and diffuse large B-cell lymphoma (Carew et al., 2019; Xu et al., 2023). In myelodysplastic syndrome (MDS) and acute myeloid leukemia (AML), DNA methyltransferase inhibitors (e.g., azacitidine), have also been tested together with HDACi, and several clinical studies have reported improved treatment responses compared with DNMT inhibition alone (Tan et al., 2014). These findings indicate that targeting complementary epigenetic mechanisms may help overcome resistance caused by redundant or compensatory regulatory pathways. In HIV-1 latency, a similar strategy combining inhibitors of histone deacetylation, repressive histone methylation, and DNA methylation may provide a more effective way to reactivate latent proviruses than targeting a single pathway alone.

**Figure 2:**
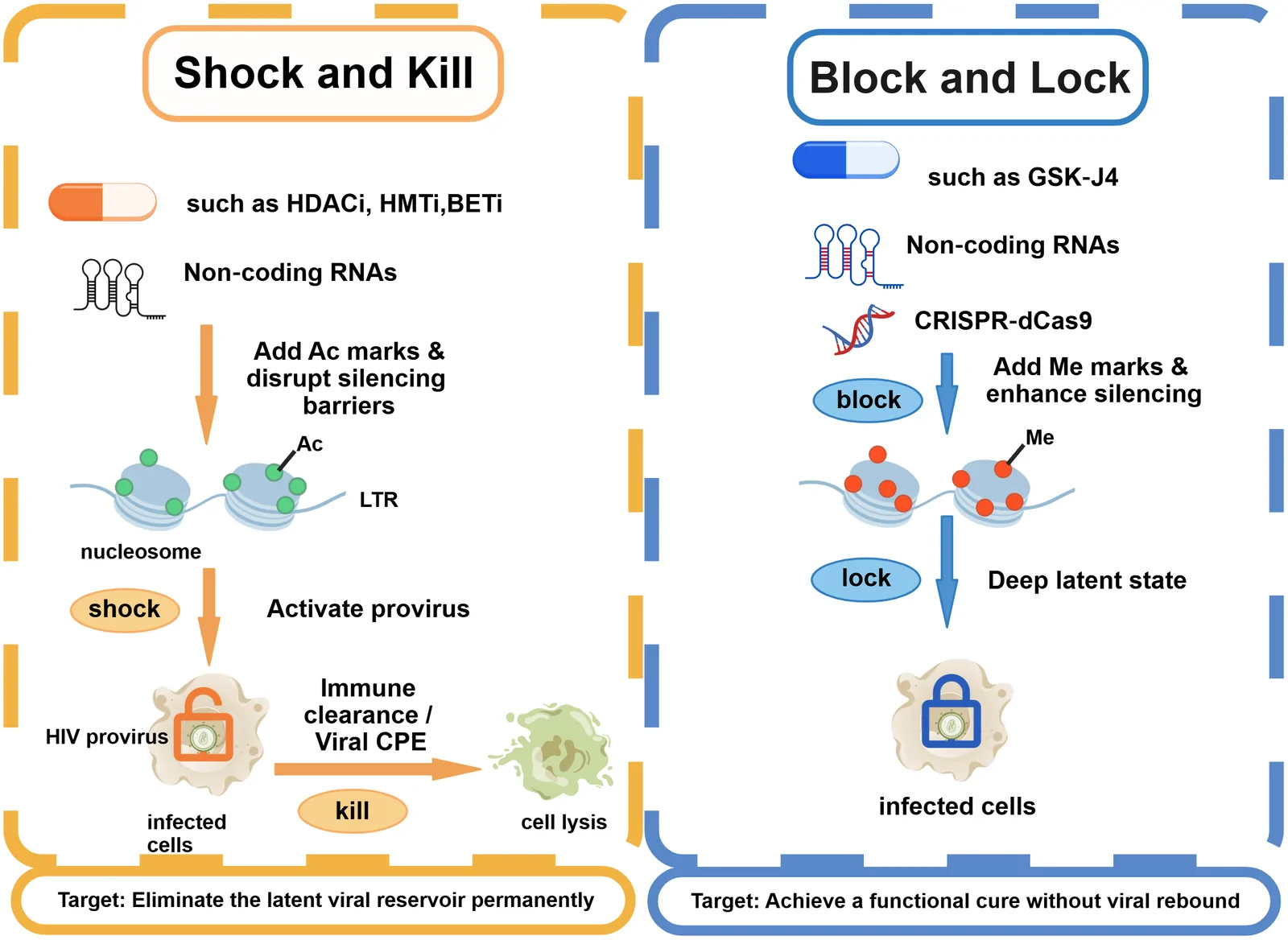
Therapeutic strategies targeting the HIV-1 latent reservoir based on epigenetic regulatory mechanisms. Therapeutic strategies targeting the HIV-1 latent reservoir based on epigenetic regulatory mechanisms. Left panel: The “shock and kill” strategy employs latency−reversing agents such as HDACi, HMTi, BETi and non−coding RNAs to add acetylation marks (Ac) and disrupt silencing barriers at the LTR, thereby activating proviral transcription. The reactivated infected cells are removed by either host’s immune system or viral cytopathic effects (CPE), leading to cell lysis and thereby permanently deplete the latent reservoir. Right panel: The “block and lock” strategy uses various methods such as inhibitor GSK-J4, non−coding RNAs, and epigenetic editing through CRISPR-dCas9 to add methylation marks (Me) and enhance silencing, driving the provirus into a deep latent state. This approach aims to achieve a functional cure by preventing viral rebound without eliminating the infected cells. This figure is generated using Generic Diagramming Platform (GDP, https://BioGDP.com) (Jiang et al., 2025).

**Table 2:** Small-molecule strategies for HIV-1 latency control: mechanisms, development status, and limitations.

Strategy and small molecule(s)	Mechanism of action	Current development status	Known limitations
Shock and kill: HDACi (vorinostat/SAHA, panobinostat, romidepsin, chidamide, CC-4a)	Increase histone acetylation and LTR accessibility; CC-4a also promotes NF-kappaB pathway activation (Archin et al., 2012; Ning et al., 2012; Elliott et al., 2014; Rasmussen et al., 2014; Søgaard et al., 2015; Kuai et al., 2018; Lu et al., 2021; McMahon et al., 2021; Wu et al., 2024).	Vorinostat, panobinostat, romidepsin and chidamide have entered clinical latency-reversal studies; CC-4a remains preclinical (Archin et al., 2012; Ning et al., 2012; Elliott et al., 2014; Rasmussen et al., 2014; Søgaard et al., 2015; Kuai et al., 2018; Lu et al., 2021; McMahon et al., 2021; Wu et al., 2024).	Induce HIV transcription without consistent reservoir reduction; dose-limiting toxicity and possible T-cell functional impairment require monitoring (Archin et al., 2012; Ning et al., 2012; Elliott et al., 2014; Rasmussen et al., 2014; Søgaard et al., 2015; Walker-Sperling et al., 2016; Kuai et al., 2018; Zhao et al., 2019; Lu et al., 2021; McMahon et al., 2021; Wu et al., 2024).
Shock and kill: HMT inhibitors (DZNep, EED inhibitors, G9a inhibitor BIX01294)	Reduce PRC2/EZH2-, EED- or H3K9 methyltransferase-mediated repressive marks at the LTR (Bouchat et al., 2012; Sharma et al., 2020; Turner et al., 2020).	Mainly preclinical or *ex vivo* studies using latency models and resting CD4^+^ T cells (Bouchat et al., 2012; Sharma et al., 2020; Turner et al., 2020).	Redundant HMT pathways and broad host-chromatin effects limit single-agent efficacy and specificity.
Shock and kill: BET/BRD9-targeting agents (JQ1, I-BRD9, dBRD9)	JQ1 relieves BRD4-mediated repression and synergizes with HDACi; BRD9 inhibitors/degraders disrupt ncBAF-associated latency maintenance (Filippakopoulos et al., 2010; Zhu et al., 2012; Boehm et al., 2013; Darcis et al., 2015; Laird et al., 2015; Luk et al., 2025).	Predominantly preclinical; BRD9 targeting has been tested in T-cell lines, primary resting memory CD4^+^ T cells and PBMCs from PLWH (Filippakopoulos et al., 2010; Zhu et al., 2012; Boehm et al., 2013; Darcis et al., 2015; Laird et al., 2015; Luk et al., 2025).	BET/BRD9 proteins regulate host transcription broadly; cytotoxicity, selectivity and *in vivo* delivery remain unresolved.
Shock and kill: m^6^A modulators (ALKBH5 inhibition)	Elevates RNA m^6^A and potentiates romidepsin-mediated latency reversal by enhancing viral RNA expression (Ali et al., 2025).	Preclinical cell-model evidence, with broader m^6^A inhibitor development informed by METTL3/epitranscriptomic studies (Yankova et al., 2021; Fiorentino et al., 2023; Guirguis et al., 2023; Harrahill and Hadden, 2024; Ali et al., 2025; Mishra et al., 2026).	m^6^A effects are transcript-specific and bidirectional; host immune RNA homeostasis may be affected.
Shock and kill: crotonylation-targeting agents (ACSS2 modulation; HDAC3/citarinostat)	Increase LTR histone crotonylation or block decrotonylation to facilitate proviral reactivation (Tan et al., 2011; Jiang et al., 2018; Crespo et al., 2022; Li et al., 2026).	Preclinical mechanistic studies in HIV latency models (Jiang et al., 2018; Crespo et al., 2022).	Metabolism-linked chromatin regulation may broadly alter host metabolic and transcriptional programs.
Block and lock: ZL0580 plus LEDGINs	BD1-selective BRD4 modulation suppresses HIV transcription; LEDGINs additively reinforce integration-site-associated silencing (Alamer et al., 2021; Pellaers et al., 2025).	Preclinical and humanized-mouse or *ex vivo* model evidence (Alamer et al., 2021; Pellaers et al., 2025).	Durability, dosing, off-target BET effects and clinical delivery remain to be defined.
Block and lock: GSK-J4	Inhibits UTX/KDM6A, maintains H3K27me3 at the LTR and supports a repressive bivalent state (Nguyen et al., 2021).	Preclinical and primary T-cell model evidence (Nguyen et al., 2021).	Enhances silencing but does not permanently block reactivation; off-target effects on host chromatin are possible (Nguyen et al., 2021).
Block and lock: METTL3 inhibitors (STM2457, STM3006, STC-15)	Reduce m^6^ A-dependent reactivation competence and attenuate HIV-1 latency reversal in CD4^+^ T-cell models (Yankova et al., 2021; Fiorentino et al., 2023; Guirguis et al., 2023; Harrahill and Hadden, 2024; Mishra et al., 2026).	Early preclinical epitranscriptomic intervention evidence (Yankova et al., 2021; Fiorentino et al., 2023; Guirguis et al., 2023; Harrahill and Hadden, 2024; Mishra et al., 2026).	Therapeutic direction, cytotoxicity, dosing window and durability of latency stabilization remain uncertain.
Block and lock: didehydro-cortistatin A (dCA)	Inhibits Tat-dependent transcription and promotes a deeply repressed proviral state complementary to epigenetic locking (Kessing et al., 2017; Vansant et al., 2020).	*In vivo* suppression of HIV rebound has been reported in preclinical models (Kessing et al., 2017).	Not a direct chromatin modifier; long-term resistance, delivery and combination design require further study.

One major reason why current therapeutic strategies have not achieved sufficient efficacy is that latency modulation is often evaluated by molecular readouts rather than by functional reservoir reduction. In the “shock and kill” approach, LRAs can induce HIV-1 transcription, but increased viral RNA does not necessarily result in sufficient antigen production for immune recognition or effective elimination of infected cells. Moreover, the heterogeneity of HIV-1 reservoirs, including differences in integration sites, chromatin states, clonal expansion, and immune accessibility, limits the efficacy of single epigenetic agents. For the “block and lock” strategy, maintaining durable and specific proviral silencing without affecting host gene expression remains a major challenge. Another limitation is that many studies rely on transformed cell lines or *in vitro* latency models, which cannot fully reflect the complexity of ART-suppressed individuals. These models may not capture reservoir diversity, tissue distribution, immune dysfunction, or drug accessibility *in vivo*. Future studies should therefore focus on primary CD4^+^ T-cell models, *ex vivo* reservoir assays, animal models, and clinically relevant outcomes, including reservoir reduction, delayed viral rebound, immune preservation, and host safety.

The safety of small-molecule latency-modulating agents, particularly LRAs used in “shock and kill” strategies, also needs to be considered from the perspective of immune effector-cell function. Successful latency reversal is only beneficial if reactivated infected cells can subsequently be recognized and eliminated by HIV-specific CD8^+^ T cells, NK cells, or other immune effectors. However, several LRAs have been reported to exert dose-, exposure time-, and cell-state-dependent effects on T cells. HDACi such as romidepsin or panobinostat may reduce antigen-specific CD8^+^ T-cell proliferation, cytokine production, degranulation, or killing capacity in some experimental settings, whereas PKC agonists can cause non-specific immune activation or toxicity at higher concentrations (Walker-Sperling et al., 2016; Zhao et al., 2019). Therefore, an LRA that efficiently induces viral transcription may still compromise the “kill” phase if it simultaneously impairs effector-cell viability or function. Future therapeutic development should evaluate viral reactivation and immune competence in parallel, including assays of T-cell viability, proliferation, IFN-γ/TNF-α production, CD107a degranulation, cytolytic activity, exhaustion-marker expression, and NK-cell function. Optimizing dose, timing, and washout windows, and combining LRAs with immune-preserving or immune-enhancing approaches, may be essential for improving both safety and efficacy.

In addition to conventional small-molecule combinations, targeted epigenetic tools, including CRISPR-dCas9-based systems, offer another possible direction for HIV cure research. Unlike small-molecule drugs that act globally across the host genome, locus-specific epigenetic editing may allow repressive marks to be added to or removed from the HIV-1 LTR more selectively. This approach could help reduce unwanted effects on host gene expression while producing longer-lasting changes at the proviral promoter. With further improvements in delivery methods, targeted epigenetic editing may help overcome some of the limitations associated with current LRAs and latency-silencing agents.

Looking forward, several epigenetic mechanisms that may be relevant to HIV-1 latency remain insufficiently studied. For example, histone lactylation, a recently described acylation modification linked to cellular metabolism, has been reported to regulate gene expression in macrophages and cancer cells by affecting chromatin accessibility. However, whether histone lactylation contributes to HIV-1 latency in CD4^+^ T cells or myeloid reservoirs remains unclear (Zhang et al., 2024; Bao et al., 2025). In addition, liquid-liquid phase separation (LLPS), which contributes to the organization of chromatin-associated regulatory complexes and heterochromatin domains, has recently been linked to HIV-1 transcription and latency maintenance (Wu et al., 2022). Further studies on these mechanisms, together with a better understanding of how nucleosome positioning, diverse histone modifications, DNA methylation, and non-coding RNAs act together at the HIV-1 LTR, may help identify new approaches for HIV-1 functional cure.

In conclusion, HIV-1 latency is underpinned by a sophisticated and resilient epigenetic regulatory network. Moving from mechanistic discovery to clinical benefit will require integrated strategies that combine multi-epigenetic-targeted regimens with precision delivery tools, while balancing therapeutic efficacy, target specificity, and safety. Continued exploration of the full complexity of the epigenetic landscape of HIV-1 latency will not only advance our fundamental understanding of viral-host interactions, but also pave the way for durable, clinically feasible functional cure approaches.
